# Medical Range Radiation Dosimeter Based on Polymer-Embedded Fiber Bragg Gratings

**DOI:** 10.3390/s21238139

**Published:** 2021-12-06

**Authors:** Marie-Anne Lebel-Cormier, Tommy Boilard, Martin Bernier, Luc Beaulieu

**Affiliations:** 1 Département de Physique, de Génie Physique et D’optique, Université Laval, Québec, QC G1V 0A6, Canada; marie-anne.lebel-cormier.1@ulaval.ca (M.-A.L.-C.); tommy.boilard.1@ulaval.ca (T.B.); martin.bernier@copl.ulaval.ca (M.B.); 2 Centre de Recherche sur le Cancer, Université Laval, Québec, QC G1R 3S3, Canada; 3 CHU de Québec, Université Laval et CRCHU de Québec, Québec, QC G1R 2J6, Canada; 4 Centre D’optique, Photonique et Laser (COPL), Université Laval, Québec, QC G1V 0A6, Canada

**Keywords:** radiotherapy dosimetry, fiber optic dosimetry, fiber optics sensors, radiation sensors, dosimetry

## Abstract

Fiber Bragg gratings (FBGs) are valuable dosimeters for doses up to 100 kilograys (kGy), but have hardly been used for the low-dose range of a few grays (Gy) required in medical radiation dosimetry. We report that embedding a doped silica fiber FBG in a polymer material allows a minimum detectable dose of 0.3 Gy for γ-radiation. Comparing the detector response for different doped silica fibers with various core doping, we obtain an independent response, in opposition to what is reported for high-dose range. We hypothesized that the sensor detection is based on the radio-induced thermal expansion of the surrounding polymer. Hence, we used a simple physical model based on the thermal and mechanical properties of the surrounding polymer and obtained good accordance between measured and calculated values for different compositions and thicknesses. We report that over the 4 embedding polymers tested, polyether ether ketone and polypropylene have respectively the lowest (0.056 pm/Gy) and largest sensitivity (0.087 pm/Gy). Such FBG-based dosimeters have the potential to be distributed along the fiber to allow multipoint detection while having a sub-millimeter size that could prove very useful for low-dose applications, in particular for radiotherapy dosimetry.

## 1. Introduction

Fiber Bragg gratings (FBGs) are now common in versatile sensors used in a wide range of applications in various industries mainly to measure temperature, stress or pressure since they can be distributed over km range and are invariant to electromagnetic interference. They can be used for instance to detect leaks on a pipeline, or to measure remotely the deformation of the wings of an airplane during flights [1,2]. In the past few decades, researchers have worked on adapting FBGs response for specific applications. For example, FBG-based temperature sensors have been used to measure temperature up to 1200 ${}^{\circ }C$ [3] and down to cryogenic temperature [4].

FBGs sensor has also proven to be useful dosimeters in nuclear environment where dose reach up to 100 kGy [5]. In these conditions, radiation produces damages to the fiber materials, which modifies the fiber refractive index [6]. This results in a shift of the peak reflectivity wavelength of the FBG that can be monitored with a specialized FBG interrogator and correlated with the absorbed radiation. It has also been shown that the radiation responses of the FBGs at high dose is highly dependent on the core composition [7,8,9,10]. For a delivered dose of 100 kGy from Co${}^{60}$, the largest wavelength shift ( 160 pm) is obtained with heavily germanium-doped fibers, i.e., 21 and 10 mol% GeO${}_{2}$, while the smallest shift ( 50 pm) is obtained with a pure silica core fiber [8]. The coating of the fiber also has a significant impact on the FBG response to radiation. For example, for a delivered dose of 40 kGy, the sensitivity of an uncoated FBG that initially shift by 15 pm can be increase up to 50 pm with an appropriate coating, such as ormocer [11]. FBG-based dosimeters have the advantage of being unaffected by Čerenkov effect as are the plastic scintillation-based optical fiber dosimeters [12].

To date, only a few attempts have been made to adapt FBG-based dosimeter for low-dose medical range applications (1 to 30 Gy) and it would prove very useful for radiotherapy dosimetry, with a sub-millimeter size, a real-time response and allowing, by design, multi-points detection. These characteristics could allow in vivo dosimetry, which would improve patient safety during the treatment [12]. Compared to other fiber-based dosimeter, which could also be a great candidate for in vivo dosimetry, the possibility of having a multipoint detector using FBG is a real advantage [13]. As of right now, a prototype relying on pre- and post-irradiation thermo-optic properties of FBGs, therefore preventing real-time use, has a reported a minimum detection of 0.160 Gy [14,15]. An optical-based temperature detector, which includes an FBG, was also used to measure radio-induced temperature increase without success [16]. To the best of our knowledge, these are the two attempts to adapt FBG-based dosimeter for low-dose medical range applications.

In this article, we present a real-time dosimeter based on plastic-coated FBGs producing a radio-induced Bragg wavelength shift which can measure doses down to 0.3 Gy. We compared the detector response for silica-based fibers with various core doping compositions and for silica-based fibers embedded in different plastics. We also developed a simple physical model based on the thermal and mechanical properties of the surrounding polymer to compare calculated and measured sensitivity values.

## 2. Materials and Methods

### 2.1. Theory

A FBG is a periodic or quasi-periodic modulation of the refractive index of the core of an optical fiber. When light propagates through the FBG, a narrow bandwidth of light, centered at the Bragg wavelength, is reflected due to backward constructive interference. The Bragg wavelength, $\lambda_{B}$, is expressed as

(1) $$\lambda_{B}=2n_{eff}\Lambda$$

where $n_{eff}$ is the effective refractive index of the reflected mode in the fiber and Λ is the period of the grating. A homogeneous and isotropic variation of temperature (∆T) or strain (ε) applied to a FBG produces a reflected wavelength shift ($∆\lambda_{B}$) expressed as

(2) $$\frac{∆\lambda_{B}}{\lambda_{B}}=\left(1-p_{e}\right)\varepsilon +\left(\alpha_{f}+\xi \right)∆T$$

where $p_{e}$ is the photo-elastic coefficient, $\alpha_{f}$ is the fiber thermal expansion coefficient and ξ is the thermo-optic coefficient. For a standard silica-based fiber, this equation reduces to [17]:

(3) $$\frac{∆\lambda_{B}}{\lambda_{B}}\approx 0.78\varepsilon +9.15\times 10^{-6}∆T$$

The limited temperature sensitivity of the FBG can be increased significantly by adding a coating made from a material with a higher thermal expansion coefficient, such as metal or plastic. In this case, the coating will pull on the fiber (which is resisting the expansion), such that the final expansion of the fiber will be the difference of the thermal expansion coefficient of the coating and the fiber, weighted by a coefficient taking into account the relative size and stiffness of each one. The reflected Bragg wavelength shift ($∆\lambda_{B}$) can be then express as [18]

(4) $$\frac{∆\lambda_{B}}{\lambda_{B}}=\left[\left(1-p_{e}\right)\left(\alpha_{c}-\alpha_{f}\right)\frac{A_{c}E_{c}}{A_{c}E_{c}+A_{f}E_{f}}\right]∆T_{c}+\left[\alpha_{f}+\xi \right]∆T_{f}$$

in which $\alpha_{c}$ and $\alpha_{f}$ are the coefficient of thermal expansion for the coating and for the fiber, $∆T_{c}$ and $∆T_{f}$ are the temperature variation for the coating and the fiber, $A_{c}$ and $A_{f}$ are the coating and the fiber cross-sectional areas and $E_{c}$ and $E_{f}$ are the Young’s modulus of the coating and of the fiber. If the temperature variation is induced by radiation energy deposition in coating, we can assume that ∆T=D/c where *D* is the dose and *c* is the coating mass heat capacity. In this case, we have:

(5) $$\frac{∆\lambda_{B}}{\lambda_{B}}=\left[\left(1-p_{e}\right)\left(\alpha_{c}-\alpha_{f}\right)\frac{A_{c}E_{c}}{A_{c}E_{c}+A_{f}E_{f}}\right]\frac{D}{c_{c}}+\left[\alpha_{f}+\xi \right]\frac{D}{c_{f}}$$

where $c_{c}$ and $c_{f}$ are respectively the coating and fiber mass heat capacity.

### 2.2. FBG-Based Sensor

The FBGs used in the experiment were all written in house using the femtosecond scanning phase-mask writing technique. The FBG writing setup, recently optimized to efficiently write distributed arrays of FBGs for sensing applications, is described in detail in [19]. Briefly, 35 fs pulses at 800 nm, generated from a Ti:Sapphire amplifier (Astrella, Coherent), are strongly focalized by an acylindrical lens through a phase-mask ($\Lambda_{\mathrm{PM}}=1070\;nm$) and inside the core of the optical fibers, through its protective coating.

To test different fiber compositions, 4 mm-long FBGs with a FWHM of 0.3 nm were written inside four different 125 µm fibers, listed in terms of expected sensitivities in Table 1. The choice of the first three fibers were based on [7], in which an increase of the germanium concentration in the core led to an increase in the sensitivity of the fiber at high doses. The fourth fiber, doped with cobalt, was tested to determine if its high attenuation would enhance its sensitivity to gamma rays.

**Figure 1:**
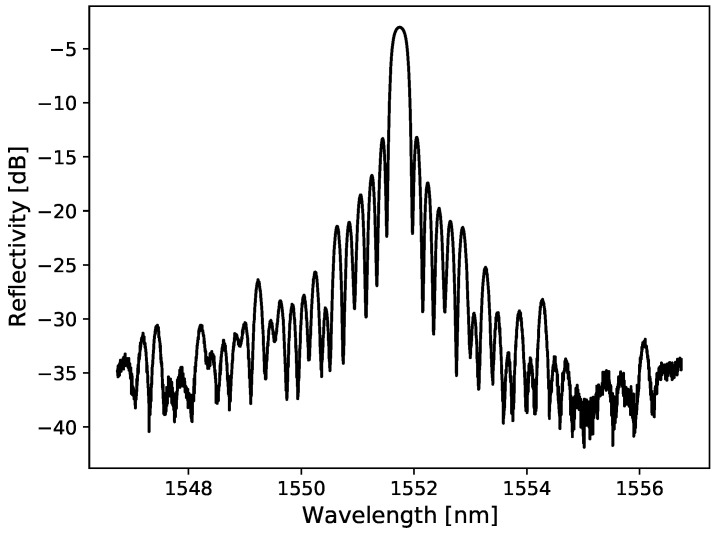
Spectrum of a 4mm-long FBG written in the LGE fiber.

To study the influence of the fibers’ composition under the same irradiation conditions, similar FBGs were written in all four fibers and were fixed using UV glue inside four closely colocated 0.20 × 1.25 × 200 mm${}^{3}$ grooves machined in a PMMA plate (5.5 × 107.5 × 205 mm${}^{3}$), which acts similar to a semi-infinite medium. A typical reflectivity spectrum written inside the LGE fiber is shown in Figure 1. All four FBGs are irradiated at the same time and monitored in real time.

**Figure 2:**
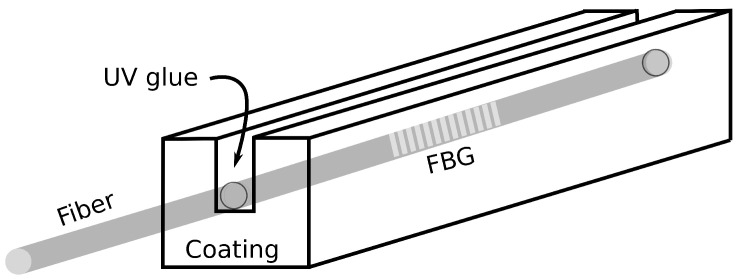
Scheme of the detector which consists of plastic-coated FBG.

To study the influence of coatings made from different materials and thickness, FBGs were written in several segments of the HGE fiber and each one was fixed with UV glue inside a square prism with a 200 µm wide groove going half of its thickness deep. A sketch of the square prisms is shown in Figure 2 for better comprehension. To study the effect of coating made of different materials, 3.0 × 3.0 × 20.0 mm${}^{3}$ square prisms made from polyether ether ketone (PEEK), polycarbonate(PC), polymethyl methacrylate (PMMA) and polypropylene (PP) were tested. Their thermal expansion coefficient $\alpha_{c}$, their specific heat capacity *c* and their Young’s modulus $E_{c}$ are listed in Table 2 and can be used with Equation (5) to test the validity of the model. Furthermore, to study the influence of coating thickness, PMMA was selected due to its ease to be machined and its availability. For that, a 2.0 × 2.0 × 20.0 mm${}^{3}$ prism was made and compared with the 3.0 × 3.0 × 20.0 mm${}^{3}$ square prism and the 5.5 × 107.5 × 205 mm${}^{3}$ PMMA plate.

### 2.3. Temperature Correction

Since the Bragg wavelength change of the FBGs is temperature dependent, it will measure the small ambient temperature variations as well as the temperature increase due to irradiation. The method that is used to correct the ambient temperature variations is to apply a linear fit to the pre- and post-irradiation data (both 60 s acquisitions) to correct the signal during the irradiation (200 s). This correction requires a linear temperature variation and is therefore not always usable. For the moment, when the temperature variation is not linear, we do not use the collected data and wait for the ambient temperature to stabilize.

### 2.4. Experimental Setup

Irradiations are performed on a Varian CLINACiX radiation therapy accelerator (Varian, CA, USA). Shifts in wavelength due to irradiation of the FBGs by a 6 MV photon beam are recorded for a dose up to 20 Gy. A 6 Gy/min dose rate and a 10 × 10 cm${}^{2}$ field size are used.

**Figure 3:**
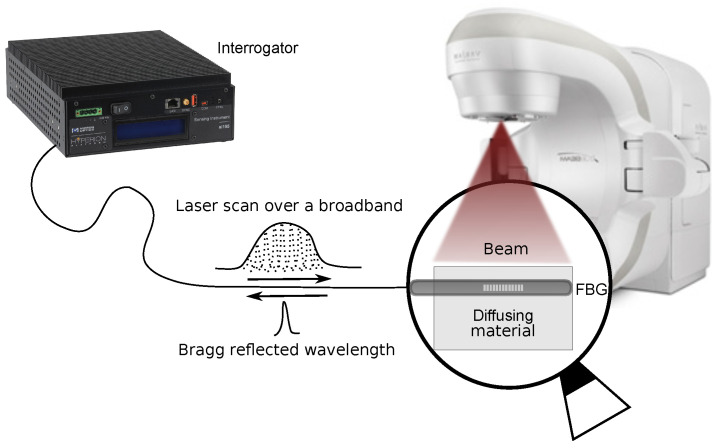
Scheme of the experimental setup. The interrogator produces the input spectrum and measures the FBG reflected spectrum, which here correspond to the signal of interest. The FBG (detector) is placed in solid water under a 6 MV clinical photon beam.

The wavelength of each FBG is recorded at 1 kHz with a commercially available 4-channel FBGs interrogator (si155, Micron Optics), with its sensing analysis software (ENLIGHT), and is averaged to have one measure per second. Assuming that the temperature variation over 1 s is negligible (0.00007 ${}^{\circ }$C), the averaging process is equivalent to measuring 1000 times the same quantity. The error of 1 pm provided by the manufacturer on every data point is then reduced by a factor 30 ($\sqrt{1000}$), which gives an error of 0.03 on the average data points. As shown in Figure 3, the detector is placed in a diffusing material (Plastic Water®, CIRS, Norfolk, VA, USA) to ensure electronic equilibrium conditions.

## 3. Results

**Figure 4:**
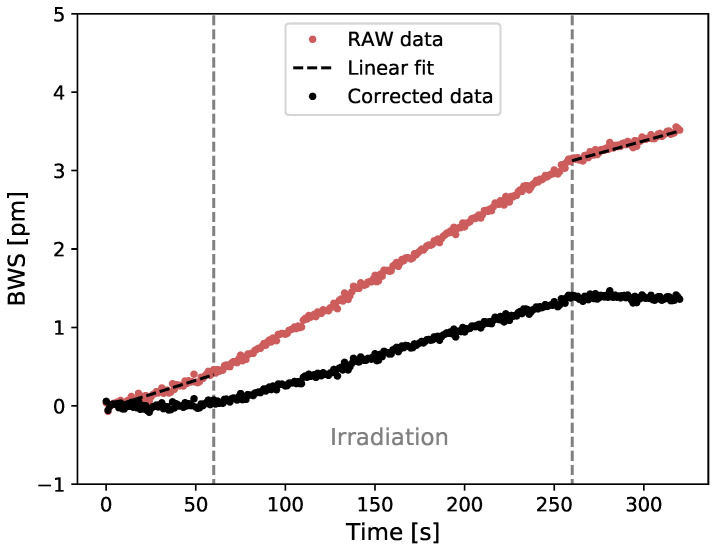
Raw and ambient temperature corrected Bragg wavelength shift (BWS) in terms of time (60 s pre- and post-irradiation) for a 20 Gy irradiation using a PMMA plate as coating (5.5 × 107.5 × 205 mm${}^{3}$) and 6 MV beam.

The raw and ambient temperature corrected Bragg wavelength shift (BWS) in terms of time are presented in Figure 4 for a delivered total dose of 20 Gy irradiation using a PMMA semi-infinite coating and 6 MV beam.

**Figure 5:**
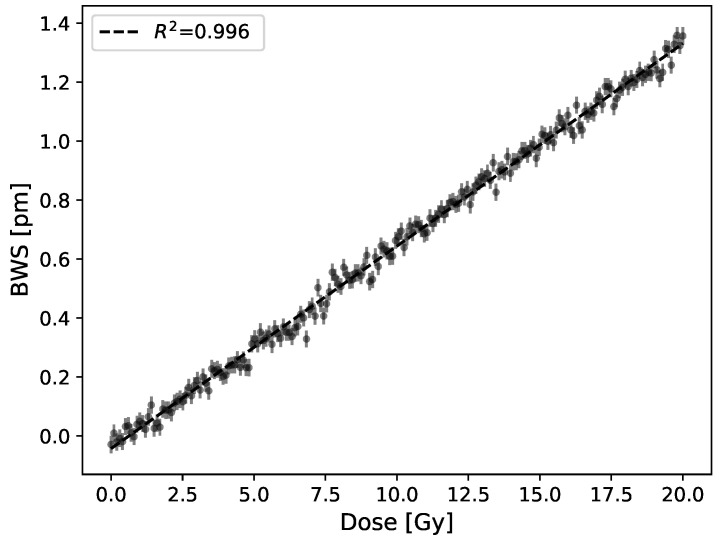
Bragg wavelength shift in terms of dose for a 20 Gy irradiation using a PMMA plate as coating (5.5 × 107.5 × 205 mm${}^{3}$) and 6 MV beam. The response to dose is 0.070 ± 0.006 pm/Gy.

A linear Bragg wavelength shift (BWS) is obtained upon irradiations using a semi-infinite PMMA coating. We performed 21 dose measurements (3 trials of 7 FBGs) and the mean signal slope is 0.070 pm/Gy with a standard deviation of 0.006 pm/Gy. The reflected wavelength shift of a FBG produced by a 20 Gy irradiation corrected for ambient temperature variations is shown in Figure 5. The standard error on data points is 0.03 pm, which, using the response to dose slopes, leads to a detection limit of this early prototype of 0.4 Gy.

**Figure 6:**
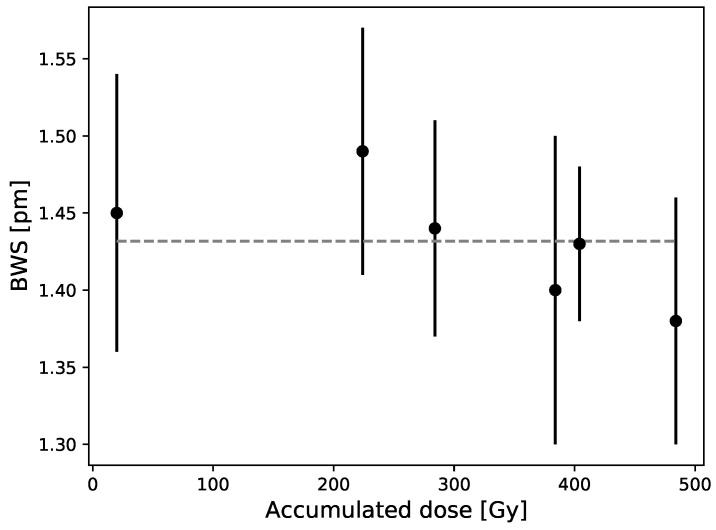
Bragg wavelength shift in terms of accumulated dose is measured throughout several months using a PMMA plate as coating (5.5 × 107.5 × 205 mm${}^{3}$) and a 6 MV beam. Each point is the mean and standard deviation of 7 FBGs shift for a 20 Gy irradiation and the dash line is the overall mean.

As shown in Figure 6, there is no significant signal variation obtained over accumulated dose for a dose up to 500 Gy. We measured the dose response of 7 FBGs over several months and obtained a mean difference and maximum difference of 2% and 4% with the overall mean (represented by the dash line).

**Figure 7:**
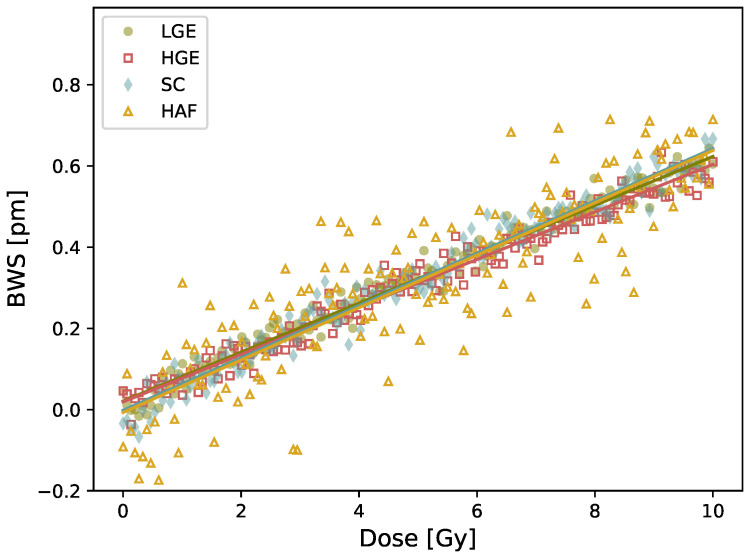
Bragg wavelength shift in terms of dose using fiber doped with germanium at low concentration (LGE), germanium at high concentration (HGE), no doping, i.e., pure silica core (SC) and cobalt (HAF) using a PMMA plate as coating (5.5 × 107.5 × 205 mm${}^{3}$) and 6 MV beam.

A similar BWS is obtained upon irradiation using detectors with different fiber compositions (Figure 7). The values and uncertainties, listed in Table 3, correspond to the mean and standard deviation on four consecutive irradiations. The dose response remains the same for all fibers. The curve is shown up to 10 Gy for clarity, but stays linear up to 20 Gy. Hence, it is impossible to modify radiation response of our detector by changing the fiber composition. Please note that the signal is noisier for the HAF fiber because the FBG inscription was harder given its lower photosensitivity, hence the Bragg peak is less defined for this fiber.

**Figure 8:**
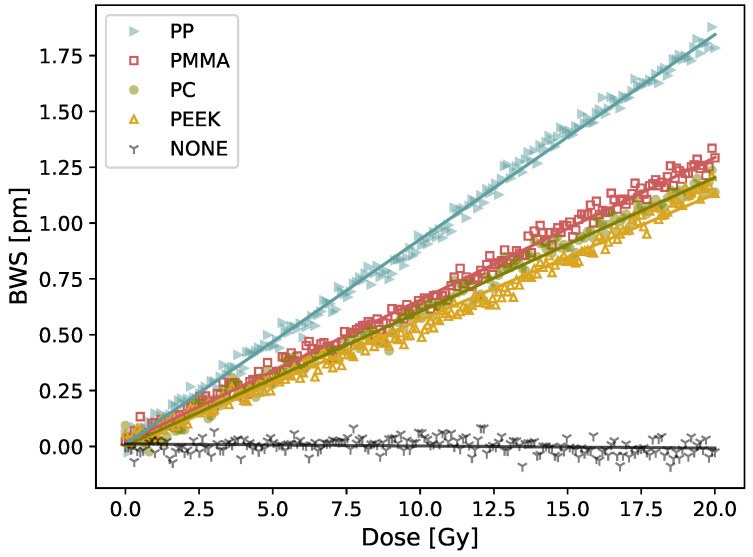
BWS in terms of dose for different coating (3 × 3 × 20 mm${}^{3}$) and a non-coated fiber (NONE). The sensitivity of each plastic used for the fiber coating is listed in Table 4.

On the contrary, using a detector with different fibers coatings changed the detector response to radiation as shown in Figure 8. The dose response and uncertainties, listed in Table 4, correspond to the mean and standard deviation on four consecutive irradiations. For all the plastics tested, a linear reflected wavelength shift is obtained. The highest response of 0.087 ± 0.001 pm/Gy is obtained using a 3 × 3 × 20 mm${}^{3}$ polypropylene (PP) coating whereas the lowest response 0.056 ± 0.008 pm/Gy is obtained using a 3 × 3 × 20 mm${}^{3}$ polyether ether ketone (PEEK) coating. For the non-coated fiber, a −0.0009 pm/Gy response is obtained, which correspond to the noise level of our detection system. The latter result is obtained by irradiating the non-coated fiber immersed in a liquid water phantom (note that fixing the fiber to solid water, made of plastic, regularly used in radiation therapy has a similar effect than using a semi-infinite coating of PMMA). The temperature increase of a plastic resulting from irradiation is dictated mainly by its expansion coefficient and its specific heat. The higher the expansion coefficient is and the smaller the specific heat is, the higher the dose response will be. From the tested materials, we can see that PP has the higher expansion coefficient and a similar specific heat than all the other plastics, which explain its stronger response to irradiation compared to the other tested plastics.

**Figure 9:**
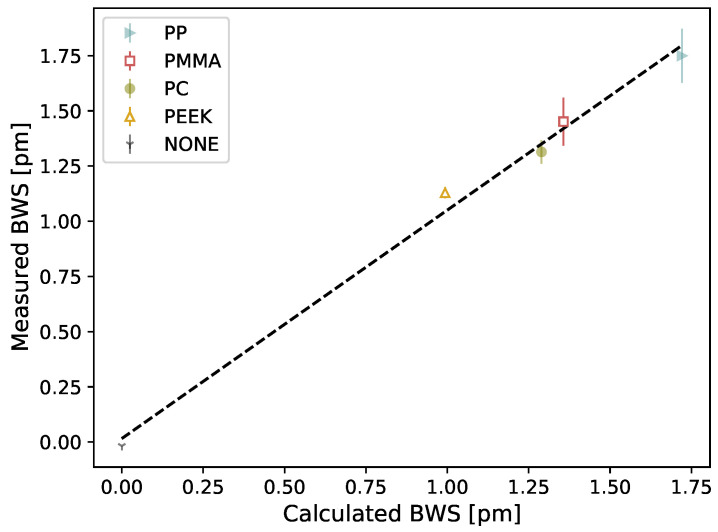
Measured BWS in terms of calculated BWS for different plastics coating with a size of 3 × 3 × 20 mm${}^{3}$ and a constant 20 Gy irradiation (y=1.03x+0.02, $R^{2}$ = 0.99). The presented values correspond to the means of four measurements and the error bar corresponds to the standard deviation. Please note that there is no error bar for the non-coated fiber data point since the measurement was achieved once due to the experimental complexity it represents.

A slope of 1.03 ($R^{2}=0.99$) is obtained when comparing the measured BWS from Figure 9 to the expected BWS calculated from Equation (5) for different plastics coating with the properties listed in Table 2. For these calculations, the Young’s modulus and cross-sectional area of the silica-based fibers are $E_{f}=72\;G\mathrm{Pa}$ and $A_{f}=\pi {\left(0.0625\right)}^{2}$${\mathrm{mm}}^{2}$, respectively [18]. Using the relation connecting dose (*D*) to temperature (∆T) from the theoretical expression: ∆T=D/c where *c* is the specific heat, the expected increase in temperature inside PMMA (for example) is 0.014 ${}^{\circ }$C for a 20 Gy irradiation.

**Figure 10:**
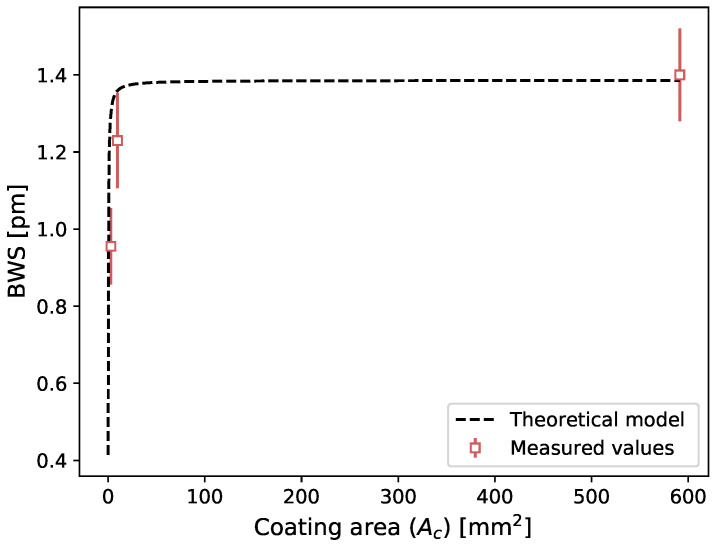
BWS in terms of coating area ($A_{c}$) for theoretical model and measured values using a 20 Gy irradiation. The presented values correspond to the means and standard deviations of three measurements, except for the 591.25 ${\mathrm{mm}}^{2}$ area (5.5 × 107.5 × 205 mm${}^{3}$ PMMA plate) which corresponds to the means of 21 measurements.

As was done previously, the wavelength shift due to the temperature variation induced by radiation beam can be calculated with Equation (5), this time for different PMMA coating sizes. The BWS in terms of coating areas for both the theoretical model and measured values are shown in Figure 10. The mean difference between the measured values and the theoretical model is 12%.

## 4. Discussion

Irradiating at low-dose FBG written in various types of fibers gave the same results (see Figure 7), which is the opposite to what is reported at large dose. For such high-dose range, changing the core or cladding composition of the fiber lead to change in the nature and concentration of radio-induced defects, and thus to different radiation responses [7,8,9].

The results presented in this study were unexpected and led us to hypothesize that the measured signal was coming from the effect of the plastic coatings on the FBG. Therefore, it was decided to perform further measurements by testing plastic coating with different thermal properties. When we changed the coating type of the FBG dosimeter, we measured different dose responses, which suggests that the response to radiation comes from the coating. Hence, our dosimeter could be working as a calorimeter. Upon irradiation, the plastic coating experience thermal dilatation, due to radio-induced temperature increase, which is measured by the FBG. The FBG-based detector would therefore measure indirectly the temperature increase generated by the dose deposition in the plastic coating. Calorimetry dosimetry is the gold standard for radiotherapy dosimetry, but is typically a substantial installation and is not easily usable in a clinical setup [21]. It is typically use to calibrate other instruments which are easier to manipulate in the clinical setting. Many attempts were made to make calorimetry possible in a clinical environment, but having a multipoint calorimeter would be unheard of. Based on the calorimetry working principle, we developed a theoretical model (Equation (5)) that show good accordance between measured and calculated values for different coating composition (slope = 1.03, R${}^{2}$ = 0.99) and for different coating sizes (mean difference of 12%). The later result is similar to the one obtained by [18]. Although further work is needed to establish that the presented approach does work as a calorimeter, the results presented are encouraging.

The 0.4 Gy detection limit obtained with the first prototype is far from the requirement of 0.01 Gy (1 cGy) for radiotherapy dosimetry [12]. Coating the dosimeter with PP allowed us to increase the dose sensitivity. The largest response to radiation, has a measured sensitivity of 0.087 pm/Gy. A standard error of 0.03 pm is measured on data points which leads to a detection limit of 0.3 Gy, which is still 30 times larger than the requirement of 0.01 Gy for radiotherapy dosimetry [12]. To reach this requirement, further optimization related to detector material and fiber size, based on our theoretical model, will be required. For example, a sensitivity increase of around 30% should be obtained by reducing the fiber size from 125 µm to 80 µm. For the detector material optimization, both the fiber and the coating may be modified. Based on the theoretical model (Equation (5)), the coating should have a thermal expansion coefficient and a Young’s modulus as high as possible while having the smallest heat capacity possible. Using an FBG interrogator with a better sensitivity could also reduce the detection limit of our sensor [22]. Another way we could increase the response to radiation would be to use a FBG written in a plastic fiber. To the best of our knowledge, this type of FBG have not used for dosimetry applications yet. The detector could also be used for small field dosimetry, where the dose per treatment is generally higher. The requirement for this type of dosimetry is a high spatial resolution, which is easily achievable with a fiber Bragg dosimeter [12].

### Limitations

It should be noted that for the calculated dose responses, we used typical values for mass heat capacity and Young’s modulus since we did not measure them for our specific samples. Therefore, the calculated values might be inexact for some plastics. The absence of uncertainties on the calculated values comes from the fact that it is hard to evaluate without measuring the mass heat capacity and the Young’s modulus. Ideally, theses parameters and the thermal expansion coefficient would be measured for each plastic, but achieving these measurements in dosimeter-like conditions (expansion rate of ∼ 10${}^{-7}$ mm/min, temperature variation of ∼ 0.01 ${}^{\circ }C$) is very challenging. The presence of UV glue is not accounted for either. In our theoretical model, we assume that the total mechanical stress produced by the polymer coating is transferred to the fiber, which might not be the case. Further testing with various UV glue with different Young’s modulus will be conducted. We also considered the expansion coefficient as isotropic.

It should also be noted that the temperature correction developed for this prototype is limiting the dosimeter to be used only for steady ambient temperature or for linear ambient temperature variations. Alternative designs bypassing this problem are being explored.

## 5. Conclusions

In this paper, we reported that embedding a silica-based FBG in a polymer material allows gamma radiation detection with a minimum detectable dose of 0.3 Gy. We obtained the same response using different silica-based fibers with various core doping compositions, but obtained a changed in the detector response for different polymer compositions and polymer sizes. Our theoretical model, based on FBG detection of radio-induced temperature increase in the coating, showed good accordance between measured and calculated values for different coating compositions and geometries. This result suggests that the response to radiation comes from the radio-induced temperature increase in the coating and therefore, our dosimeter might be working as a calorimeter. This detector shows great potential for a radiotherapy application, especially for MRI-linac since it is inherently invariant to electromagnetic interference.

## Figures and Tables

**Table 1:** Fibers used in the experiment.

Designation	Fiber Name (Manufacturer)	Description
SC	Super RadHard SMF (DrakaElite)	Acrylate-coated 8/125/242 pure silica core fiber with extremely low sensitivity to radiation
LGE	BF04446 (OFS)	Standard polyimide-coated 9/125/155 germanium-doped optical fiber
HGE	BF06160-02 (OFS)	Polyimide-coated 4.6/125/155 optical fiber with a higher germanium concentration than LGE
HAF	HAF-CMS (CorActive)	8/125 cobalt-doped fiber with an attenuation of 10 dB/cm, uncoated

**Table 2:** Coating material of the four plastics used in the experiment, and their thermal expansion coefficient ($\alpha_{c}$) (from manufacturer), specific heat capacity (*c*) and Young’s modulus ($E_{c}$) [20].

Designation	Material	$\alpha_{c}$	*c*	$E_{c}$
		$10^{-6}$${}^{\circ }$C${}^{-1}$	J/kg $\;\cdot {\;}^{\circ }$C	MPa
PP	Polypropylene	120	2000	1000
PMMA	Polymethyl methacrylate	60	1470	3200
PC	Polycarbonate	65	1700	2400
PEEK	Polyether ether ketone	55	2200	3600

**Table 3:** Dose response for different fiber core compositions using a PMMA plate as coating (5.5 × 107.5 × 205 mm^3^).

Fiber	Dose Response
	pm/Gy
LGE	0.060 ± 0.004
HGE	0.060 ± 0.003
SC	0.061 ± 0.002
HAF	0.06 ± 0.01

**Table 4:** Dose response for different coating with a size of 3 × 3 × 20 mm${}^{3}$ and a non-coated fiber (NONE).

Plastic Coating	Dose Response
	pm/Gy
PP	0.087 ± 0.001
PMMA	0.066 ± 0.003
PC	0.060 ± 0.005
PEEK	0.056 ± 0.006
NONE	−0.001 ± 0.001

## Data Availability

The data presented in this study are available on request from the corresponding author.
